# Combining Feature Extraction Methods and Categorical Boosting to Discriminate the Lettuce Storage Time Using Near-Infrared Spectroscopy

**DOI:** 10.3390/foods14091601

**Published:** 2025-05-01

**Authors:** Xuan Zhou, Xiaohong Wu, Zhihang Cao, Bin Wu

**Affiliations:** 1 School of Electrical and Information Engineering, Jiangsu University, Zhenjiang 212013, China; 3220503081@stmail.ujs.edu.cn; 2 High-Tech Key Laboratory of Agricultural Equipment and Intelligence of Jiangsu Province, Jiangsu University, Zhenjiang 212013, China; 3 Mengxi Honors College, Jiangsu University, Zhenjiang 212013, China; 3220501022@stmail.ujs.edu.cn; 4 Department of Information Engineering, Chuzhou Polytechnic, Chuzhou 239000, China; 5 School of Computer Science and Engineering, Southeast University, Nanjing 211102, China

**Keywords:** lettuce, near-infrared spectroscopy, feature extraction, classification

## Abstract

Lettuce is a kind of nutritious leafy vegetable. The lettuce storage time has a significant impact on its nutrition and taste. Therefore, to classify lettuce samples with different storage times accurately and non-destructively, this study built classification models by combining several feature extraction methods and categorical boosting (CatBoost). Firstly, the near-infrared (NIR) spectral data of lettuce samples were collected using a NIR spectrometer, and then they were preprocessed using six preprocessing methods. Next, feature extraction was carried out on the spectral data using approximate linear discriminant analysis (ALDA), common-vector linear discriminant analysis (CLDA), maximum-uncertainty linear discriminant analysis (MLDA), and null-space linear discriminant analysis (NLDA). These four feature extraction methods can solve the problem of small sample sizes. Finally, the classification was achieved using classification and regression trees (CARTs) and CatBoost, respectively. The experimental results showed that the classification accuracy of NLDA combined with CatBoost could reach 97.67%. Therefore, the combination of feature extraction methods (NLDA) and CatBoost using NIR spectroscopy is an effective way to classify lettuce storage time.

## 1. Introduction

Lettuce (*Lactuca sativ* L.), belonging to the *genus Lactuca* of the family Asteraceae, is a kind of leafy vegetable rich in vitamins, minerals, and bioactive compounds. Due to its rich nutritional value and diverse ways of consumption, it is deeply loved by consumers [1]. Lettuce contains abundant nutrients, including beta-carotene, lutein, folic acid, vitamins C and E, dietary fiber, trace elements (potassium and calcium), etc. [2]. Consuming lettuce has healthy effects such as anti-aging, lowering cholesterol, preventing cancer, and promoting blood circulation [2]. Studies have proved that the lettuce storage time was an important influencing factor in determining its nutritional value [3]. For example, if stored for too long, the lettuce will undergo browning and lignification, which will affect the eating quality and nutritional value of the lettuce [4]. However, when consumers purchase lettuce in their daily lives, they cannot accurately determine how long it has been stored. Currently, there are no labels indicating the actual storage situation on the market for sale, so it is impossible to judge the quality of lettuce. Therefore, to protect the interests of consumers and ensure healthy consumption, it is crucial to develop a method that can quickly, accurately, and non-destructively identify the lettuce storage time.

In recent years, many researchers have proposed methods for identifying the lettuce storage time. Meng et al. analyzed the metabolites in lettuce by combining ultra-high-performance liquid chromatography–quadrupole electrostatic field orbital-ion-trap mass spectrometry (UHPLC-Q-Orbitrap-MS) with gas chromatography–mass spectrometry (GC-MS) and effectively judged the freshness and storage time of lettuce [5]. Wu et al. accurately identified the lettuce storage time by combining near-infrared (NIR) spectroscopy with principal component analysis (PCA), linear discriminant analysis (LDA), and generalized fuzzy k-harmonic means (GFKHM) clustering [6]. Hu et al. proposed a novel method for identifying the lettuce storage duration, which combines NIR spectroscopy and fuzzy uncorrelated QR analysis (FUQRA) to construct a classification model. This approach achieves non-destructive, rapid, and accurate identification of the lettuce storage time [7]. Although these methods can all achieve the classification of lettuce storage times, the feature extraction method for NIR spectra of lettuce is a two-stage method, such as PCA and LDA. However, due to the neglect of the inter-relationships among data points and the category information, PCA always causes discriminant information loss [8].

NIR spectroscopy is a spectroscopic analysis technology that boasts advantages such as rapidity, non-destructiveness, and low cost. In recent years, it has been widely applied in some fields, including agriculture [9,10,11], food [12,13,14,15,16], medical/clinical [17,18], and life sciences [19,20], especially in the field of food quality analysis and detection, where NIR spectroscopy holds significant influence [21,22,23,24,25,26,27]. For instance, Shi et al. obtained the hyperspectral image data of yellow peach samples through the NIR hyperspectral imaging system and processed the sample data using multi-objective feature selection and multi-task models, achieving precise detection of the storage conditions and storage time of yellow peaches [28]. Yao et al. proposed a model population analysis to reduce the dimensionality of NIR spectra and established a prediction model for the S-ovalbumin content of eggs. This model showed good performance in the detection of the freshness of eggs at different stages [29]. Vitalis et al. used NIR spectroscopy coupled with PCA, LDA, and partial least-squares regression (PLSR) to accurately and non-destructively detect the freshness of lettuce during refrigeration [3]. Ding et al. combined a Support Vector Machine (SVM) with the Comprehensive Learning Particle Swarm Optimization (CLPSO) algorithm to construct the CLPSO-SVM classification model. They also combined it with NIR spectroscopy to achieve accurate classification of the different qualities of Huangshan Maofeng tea [30].

Due to the characteristics of the collected NIR spectra, such as a low signal-to-noise ratio, severe spectral band overlap, and high dimensionality, direct classification of the original spectra is not feasible, and the classification accuracy is relatively low. Spectral preprocessing and feature extraction can be used to solve the above problems. Spectral preprocessing is of great significance in smoothing the spectral data and enhancing the spectral resolution. Currently, spectral preprocessing methods include Savitzky–Golay (SG) filtering [31,32], standard normal variate (SNV) [33], multiplicative scatter correction (MSC) [34], etc., as well as combinations and complementarities between single preprocessing methods [35,36,37]. After the original spectra are preprocessed, feature extraction processing is carried out. The most commonly used feature extraction algorithm is LDA, which can effectively reduce the dimensionality of spectral data and extract effective classification information [38]. However, when using LDA to process the high-dimensional NIR spectra directly, the small sample size (SSS) problem often occurs [39]. That is, when the number of experimental samples is much smaller than the feature dimension of sample data, the intra-class scatter matrix $S_{W}$ in the LDA will have a singularity. So, $S_{W}^{-1}$ cannot be solved, resulting in the inability to compute the optimal projection direction. To solve the SSS problem, researchers have improved the LDA algorithm and proposed many new methods, such as approximate linear discriminant analysis (ALDA). ALDA uses $S_{a}^{-1}$ as an approximation of $S_{W}^{-1}$ through singular value decomposition (SVD) and then computes the optimal direction matrix *w* through $S_{a}^{-1}$ to achieve dimensionality reduction [40]. Besides ALDA, there are also common-vector linear discriminant analysis (CLDA) [41], maximum-uncertainty linear discriminant analysis (MLDA) [42], and null-space linear discriminant analysis (NLDA) [43]. All these methods can achieve feature extraction for the small sample size of NIR spectral data.

After preprocessing and feature extraction for dimension reduction, the spectral data need to be classified in combination with classifiers. In recent years, the classification and regression tree (CART) algorithm has been frequently adopted by researchers. This is due to its simplicity and efficiency in performing classification tasks. It makes use of the characteristic of decision trees that they can automatically capture the complex relationships among features in sample data to achieve precise classification. Hansen et al. for the first time utilized the CART algorithm on the physicochemical data of dairy products to achieve the detection of adulteration in milk samples [44]. However, since CART is a single decision tree algorithm, it is prone to overfitting when processing complex datasets. The categorical boosting (CatBoost) algorithm has greatly enhanced the generalization ability of the classification model by iteratively training multiple weak decision trees to obtain a strong classifier. Meanwhile, the CatBoost algorithm can also achieve efficient and accurate classification for complex datasets. Gao et al. have combined Autoencoder with the CatBoost algorithm to construct the Autoencoder–CatBoost model, achieving non-destructive detection of the quality of Yunling snowflake beef [45].

In this study, firstly, the spectral data of lettuce with different storage times were collected using an NIR spectrometer. Then, six different preprocessing methods were applied to preprocess the NIR spectra. After that, four feature extraction algorithms were used to extract features from the sample data. Finally, two classification algorithms were employed to classify the data after dimension reduction. Through the above steps, this study aims to build a classification model combining feature extraction and CatBoost, which is to achieve non-destructive, rapid, and accurate identification of Italian all-year-round bolt-resistant lettuce storage times.

## 2. Materials and Methods

### 2.1. Sample Cultivation and Preservation

The samples used in this experiment were Beishan No.3 lettuces. They were cultivated in a Venlo-type greenhouse (Laboratory Venlo of Modern Agricultural Equipment of Jiangsu University, Zhenjiang, China). The cultivation method adopted was perlite bag culture. During the cultivation process, the greenhouse environmental temperature was maintained at about 10 to 33 °C, and the relative humidity was kept at about 30% to 90% RH.

When the lettuce samples reached the rosette stage, 60 fresh samples were collected. The samples were thoroughly washed and placed in labeled and sealed food preservation bags and then stored in a refrigerator at 4 °C. The laboratory temperature was maintained at around 15 °C, and the relative humidity was kept at approximately 70% to ensure the lettuce quality when collecting NIR spectra. During the process of spectral acquisition, no chemical or physical treatment was applied to the lettuce samples.

### 2.2. NIR Spectrum Collection and Software Tools

The Antaris II NIR spectrometer (Thermo Fisher Scientific Co., Waltham, MA, USA) was used for the acquisition of NIR spectra of lettuce. Before the acquisition, the NIR spectrometer was preheated for 1 h. During the acquisition process, the spectrometer was adjusted to the reflectance-integrating sphere mode. The spectral scanning range was 4000 cm^−1^ to 10,000 cm^−1^, with a scanning interval of 3.856 cm^−1^. Each sample was scanned 32 times to obtain the average value of the diffuse reflectance spectra. To ensure the smallest possible error, each sample was sampled three times, whose average value was taken as the final sampling data. The spectral collection samples were 60 complete lettuce leaves. NIR spectra of the whole leaves were collected every 6 h and stored in the refrigerator after collection. They were taken out for the next collection when needed, and this process was repeated 5 times. Each collection was the same batch of lettuce samples, with only the storage time difference. A total of 300 NIR spectra were obtained, each of which has a feature dimension of 1577. Based on the collection time, the collected NIR spectroscopy data were classified into five categories: 0 h, 6 h, 12 h, 18 h, and 24 h. The number of samples in each category was 60. The dataset in this experiment was divided using five-fold cross-validation. That is, the 60 samples of each category were divided into five parts. In each fold, one part of the samples from each category was taken as the test set, and the other four parts were used as the training set. Thus, five different test set–training set combinations were obtained. In each fold, the test set contained 60 samples, and the training set contained 240 samples. The training set in each fold was used for model training, and the test set was used for model performance evaluation. Finally, the five model evaluation results were combined to obtain the final model performance evaluation result.

The software utilized in this study is MATLAB R2018a (The Mathworks Inc., Natick, MA, USA) and Python 3.11.5 for processing and analyzing the NIR spectra. Moreover, all the classification models proposed in this paper are self-built.

### 2.3. Preprocessing Methods

During the process of collecting NIR spectra of lettuce samples, some external noises may interfere, resulting in the low classification accuracy of the NIR spectra. Moreover, due to the influence of the physical properties of the samples themselves, such as rough surfaces, uneven particle distribution, etc., light scattering effects also occur, which may interfere with the NIR spectra. At the same time, the stability of the instruments in the laboratory and changes in the state of the samples themselves may also cause baseline drift in the spectra, thereby affecting the overall position shift of the spectra. Based on the above problems, to ensure the accuracy of classification, preprocessing methods need to be carried out on the original NIR spectra [46,47]. In this study, six different preprocessing methods were performed for processing the NIR spectra, including SG, SNV, MSC, and three combination methods: MSC + SG, SNV + SG, and SNV + MSC.

SG can effectively reduce noise and interfering signals in the spectrum, enhance the signal-to-noise ratio of the data, make the spectral curve smoother, and play roles such as separating overlapping spectral peaks and eliminating spectral baseline drift [30,31]. SNV can effectively eliminate the influence of light scattering caused by the physical properties of the sample itself and can also increase the stability of spectral data [27]. MSC can also effectively eliminate light scattering and noise caused by the surface particle size, roughness, and optical path variation of the sample itself [34]. MSC + SG, SNV + SG, and SNV + MSC can comprehensively improve the spectral information over a single preprocessing method [35,36]. The above six preprocessing methods have effectively improved the NIR spectra, such as the low signal-to-noise ratio, scattering influence, and baseline drift in the original spectra.

### 2.4. Feature Extraction Algorithms

Since the spectral dimensionality of the samples in this experiment is much larger than the sample number, the SSS problem often occurs, which makes it impossible to run the LDA algorithm because of the singularity of the intra-class scatter matrix. To address the SSS issue that arises during the feature extraction, in this experiment, four improved linear discriminant analysis algorithms, namely ALDA, CLDA, MLDA, and NLDA, were performed to extract features from NIR spectra of lettuce samples.

#### 2.4.1. Approximate Linear Discriminant Analysis

ALDA is an improved LDA method for solving the SSS problem. The core idea of this algorithm is to replace the original within-class scatter matrix $S_{w}^{-1}$ with the approximate matrix $S_{a}^{-1}$, avoiding the problem of singularity that makes it impossible to compute the inverse matrix [40]. ALDA can retain the null space and range space of the within-class scatter matrix $S_{w}$ and improve the classification accuracy. The steps of ALDA are described below [40]:

Step 1: Preprocessing: Perform singular value decomposition on the matrix $A_{t}$ to obtain the eigenvectors $U_{t}$, and then project the data sample X onto the range space of $S_{T}$ to achieve dimensionality reduction, that is $Y=U_{t}^{T}X$.
Step 2: Calculate ${\hat{S}}_{W}$ and ${\hat{S}}_{B}$, and obtain the eigenvector $U_{W}$ and the square root of the eigenvalue $D_{W}$ of ${\hat{S}}_{W}$ through singular value decomposition.
Step 3: Calculate the maximum value $\alpha =\max(diag(D_{W}))$ of the square root of the eigenvalue of ${\hat{S}}_{W}$, and define the approximate matrix ${\hat{S}}_{\alpha }^{-1}=U_{W}D_{\alpha }^{2}U_{W}^{T}$ to replace ${\hat{S}}_{W}^{-1}$.
Step 4: Solve the eigenvalue problem ${\hat{S}}_{\alpha }^{-1}{\hat{S}}_{B}w_{i}=\lambda_{i}w_{i}$ to obtain the optimal projection direction matrix W.

In Steps 1 and 2, $S_{T}$ represents the total scatter matrix before dimensionality reduction; Y is represented as the data matrix obtained by projecting the original data sample X onto the range space of $S_{T}$; ${\hat{S}}_{W}$ is the scatter matrix within classes after dimensionality reduction; and ${\hat{S}}_{B}$ is the scatter matrix between classes after dimensionality reduction.

#### 2.4.2. Common-Vector Linear Discriminant Analysis

CLDA is an improved method for LDA based on the core idea of common vectors. It calculates common vectors in the null space of the within-class scatter matrix and finds the optimal projection direction vector through common vectors, avoiding the SSS problem caused by the singularity of the within-class scatter matrix that makes a direct solution impossible [41]. Compared with LDA, CLDA reduces a large amount of calculation, solves the SSS problem, and improves the data recognition accuracy. The steps of CLDA are described below [41]:

Step 1: Calculate the $S_{W}$, and then calculate the non-zero eigenvalues and corresponding eigenvectors of $S_{W}$ through matrix $AA^{T}$. These eigenvectors constitute the projection vectors Q of the $S_{W}$ range space.
Step 2: Select the sample from each category and project it onto the null space of $S_{W}$ to obtain $x_{com}^{i}$.
Step 3: Calculate $S_{com}$ and matrix $A_{com}A_{com}^{T}$ to obtain the non-zero eigenvalues and corresponding eigenvectors of $S_{com}$. These eigenvectors are then used as the optimal projection vector $w_{k}$, which serves as the discriminant common vector.

In the above steps, $S_{W}$ represents the within-class scatter matrix; $x_{com}^{i}$ denotes the common vector of the i-th class; $S_{com}$ represents the within-class scatter matrix of the total common vectors.

#### 2.4.3. Maximum-Uncertainty Linear Discriminant Analysis

MLDA is a new LDA method based on the modified within-class scatter matrix $S_{W}$. Its main idea is to expand the eigenvalues of the merged covariance matrix $S_{P}$ that replaces the original within-class scatter matrix $S_{W}$, thereby creating a new within-class scatter matrix to avoid the problem of singularity [42]. MLDA not only solves the SSS problem but also improves the stability of the within-class scatter matrix and expands the application scope. The steps of the MLDA are described below [42]:

Step 1: Calculate $S_{W}$ and $S_{P}$, and obtain the eigenvalues and eigenvectors of $S_{P}$.
Step 2: Calculate $\bar{\lambda }$ through the $S_{P}$ eigenvalue calculation.
Step 3: Compare the original eigenvalue $\lambda_{i}$ with $\bar{\lambda }$. If it is greater than $\bar{\lambda }$, it will be retained; if it is less than $\bar{\lambda }$, it will be replaced by $\bar{\lambda }$ to form a new diagonal matrix $\Lambda^{*}$.
Step 4: Construct $S_{W}^{*}$ using the diagonal matrix $\Lambda^{*}$ of the new eigenvalues, and replace $S_{W}$ with $S_{W}^{*}$. Then, substitute this into the Fisher criterion of LDA [48] to obtain the optimal projection direction.

In the above steps, $S_{W}$ represents the original within-class scatter matrix; $S_{P}$ is the merged covariance matrix; $\bar{\lambda }$ is the average eigenvalue of $S_{P}$; $\Lambda^{*}$ is the modified eigenvalue matrix; $S_{W}^{*}$ is the modified within-class scatter matrix of the new class.

#### 2.4.4. Null-Space Linear Discriminant Analysis

NLDA is an improved LDA method based on the null space of $S_{w}$. It eliminates the sample category differences by projecting the sample data onto the null space of $S_{w}$ and only requires solving the eigenvalues and eigenvectors of $S_{b}$, avoiding the inability to solve the inverse matrix of $S_{w}$ due to the singularity [43]. This method not only solves the SSS problem but also reduces the computational load and improves the recognition accuracy. The steps of the NLDA are described below [43]:

Step 1: Calculate the $S_{w}$ and $S_{b}$ of the sample data.

Step 2: Determine the magnitude relationship between the rank r and the dimension n of $S_{w}$. If r=n, substitute it into the Fisher discriminant criterion and solve for the maximum eigenvalue of ${\left(S_{b}+S_{w}\right)}^{-1}S_{b}$ and its corresponding eigenvector. If r<n, proceed to the next steps.

Step 3: Calculate $S_{w}=U\Sigma V^{T}$ through singular value decomposition. Since $S_{w}$ is symmetric, U=V.

Step 4: Extract matrix Q from matrix V for the purpose of projecting onto the null space of $S_{w}$.

Step 5: Project $S_{b}$ onto the null space of $S_{w}$ to obtain ${\tilde{S}}_{b}$.

Step 6: Solve the eigenvalues and corresponding eigenvectors of ${\tilde{S}}_{b}$, and select the eigenvector corresponding to the largest eigenvalue as the optimal discriminant vector set.

In the above steps, $S_{w}$ represents the intra-class divergence matrix; $S_{b}$ represents the inter-class divergence matrix; V represents the singular vector matrix; Q represents the basis vector of the null space of $S_{w}$; and ${\tilde{S}}_{b}$ represents the inter-class divergence matrix projected onto the null space of $S_{w}$.

### 2.5. Classifiers

After extracting the features from the NIR spectra, classifiers need to be adopted to classify the data after dimension reduction. In this study, two classifiers were used, namely CART and Catboost.

#### 2.5.1. Classification and Regression Trees

CART is a supervised learning algorithm based on a binary tree, being widely used in classification tasks [49]. Its core idea is to construct a binary classification tree through binary recursive splitting. During the process of constructing a classification tree, CART uses the Gini index as the basis for node splitting; the formula for the Gini index is as follows [49]:

(1) $$G\mathrm{ini}(D)=\sum_{k=1}^{n}p_{k}(1-p_{k})=1-\sum_{k=1}^{n}p_{k}^{2}$$

where D indicates the dataset, n represents the number of sample categories, and $p_{k}$ denotes the probability that a sample point belongs to the k-th category. The Gini index can measure the purity of the distribution of the sample dataset. The smaller the Gini index is, the more concentrated the distribution of sample dataset categories is.

When dividing the dataset, the Gini index of each feature at this node is calculated; that is, for each feature, the dataset is divided into two subsets, respectively, and the Gini index of each subset after the division is calculated. Then, by weighted calculation, the Gini index corresponding to this feature can be obtained. After that, the feature with the smallest Gini index as the splitting node is selected. For the subsets obtained after the division, the above steps are repeated via a recursive call. The splitting stops until the Gini index of all features in the sub-nodes is less than the splitting threshold, and then a binary decision tree suitable for classification can be obtained [49].

After obtaining the decision tree, the CART algorithm will also apply the Cost Complexity Pruning (CCP) method to prune the decision tree to avoid overfitting problems. It minimizes the loss function of the subtree. The loss function is composed of the prediction error of the subtree and regularization, and the size of the loss function is determined by the number of leaf nodes of the subtree. This algorithm achieves the minimization of the loss function by pruning unnecessary subtrees. However, how to choose the branches to be pruned is a very important issue. Therefore, this algorithm introduces a pruning threshold $\beta_{h}$, that is [49]:

(2) $$\beta_{h}=\frac{R(t)-R(T_{t})}{\left|N_{T_{t}}\right|-1}$$

where R(t) represents the misclassification cost of the node t after pruning the subtree $T_{t}$, $R(T_{t})$ represents the misclassification cost of the subtree $T_{t}$ without pruning, and $\left|N_{T_{t}}\right|$ is the number of leaf nodes of the subtree $T_{t}$. The minimum pruning threshold $\beta_{\min}$ can be obtained by calculating and comparing the pruning thresholds of each node. Then, by visiting the decision tree nodes from bottom to top, the nodes with the pruning threshold equal to $\beta_{\min}$ are pruned. After pruning, the subtree $T_{0}$ is obtained. The above operation is continued on the pruned decision tree $T_{0}$ until the tree consists only of the root node. Eventually, an optimal subtree set $\left\{T_{0},T_{1},\ldots ,T_{n}\right\}$ is obtained. Then, the optimal subtree set is evaluated through cross-validation to obtain the best classification model.

#### 2.5.2. Categorical Boosting

**Figure 1:**
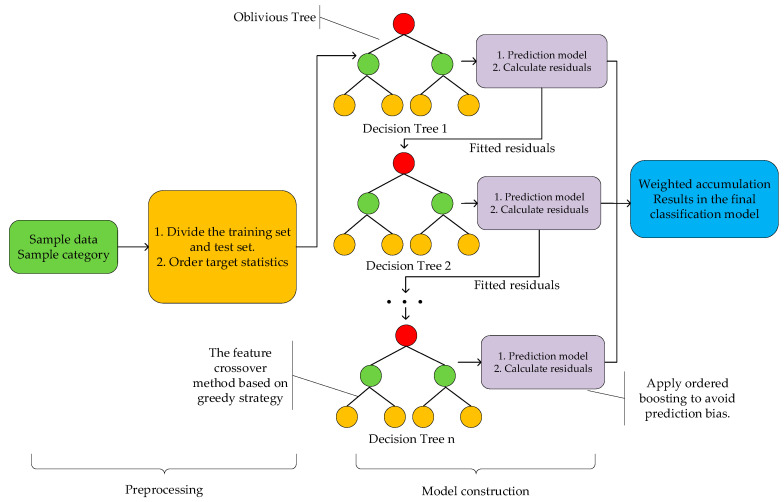
The CatBoost schematic diagram.

CatBoost is an ensemble learning model using a gradient-boosting decision tree (GBDT) [50], which can handle categorical judgment tasks quickly and efficiently. The basic idea of this algorithm is to train multiple weak classifiers iteratively, where the weak classifiers are usually oblivious decision trees. Each new weak classifier aims to correct the errors of the previous classifier to ultimately generate an efficient and powerful strong classifier. The CatBoost schematic diagram is shown in Figure 1. CatBoost first preprocesses the dataset to distinguish the training set from the test set and convert categorical features into numerical forms. Then, it builds weak classifiers based on symmetrical binary decision trees, calculates the residuals of the weak classifiers, and constructs a new decision tree to fit the residuals of the previous tree. This process repeats until the preset number of iterations is reached. Finally, the predicted values of all base classifiers are summed up to build a powerful classification model.

It can be seen that compared with the traditional GBDT algorithm, the CatBoost algorithm has made some improvements. For instance, it uses the ordered target statistics method in the preprocessing to convert categorical features into numerical ones, and the numerical formula is as follows [50]:

(3) $${\hat{x}}_{k}^{i}=\frac{\sum_{x_{j}\in D_{k}}[x_{j}^{i}=x_{k}^{i}]\cdot y_{j}+ap}{\sum_{x_{j}\in D_{k}}[x_{j}^{i}=x_{k}^{i}]+a}$$

In the formula, ${\hat{x}}_{k}^{i}$ represents the encoded value of the i-th categorical variable in the k-th sample; $x_{j}$ is the j-th sample; $x_{j}^{i}$ represents the value of the i-th categorical variable in the j-th sample; $D_{k}$ is the dataset related to sample k; [] is the indicator function; $y_{j}$ is the target variable value of the j-th sample; a is the smoothing coefficient; and p is the prior value, usually the global mean of the sample. During the encoding process, CatBoost employs an ordered boosting method, meaning that each sample is only calculated based on the samples ranked before it, effectively avoiding the problem of target leakage. Additionally, a smoothing term is added to prevent overfitting. The ordered target statistics not only solve the problem of feature number explosion that occurs when using one-hot encoding but also significantly improve the efficiency of processing categorical features.

During the process of constructing decision trees, since the numerical features can lead to ineffective feature crossing, CatBoost also adopts a feature combination method based on a greedy strategy. Specifically, it does not consider feature crossing during the first split of the decision tree. However, it forms crossings between the current feature and all the features split previously in each subsequent split. This approach enables dynamic capture of the relationships between features while avoiding the explosive growth of feature dimensions.

During the model construction, CatBoost also adopts the ordered boosting method; that is, when training each sample, it only considers the samples ranked before the current sample, thereby avoiding the problem of target leakage that occurs in a GBDT when each tree fits the residuals of the previous tree. Moreover, it reduces prediction bias and enhances the model’s generalization ability.

Through the aforementioned improvement measures, CatBoost can complete classification tasks more quickly and effectively and enhance the stability and prediction efficiency of the classification model. Especially when dealing with datasets containing a large number of categorical features, CatBoost is significantly superior to a GBDT. As a result, it has been widely applied by researchers in classification prediction tasks.

## 3. Results

### 3.1. NIR Spectral Analysis

**Figure 2:**
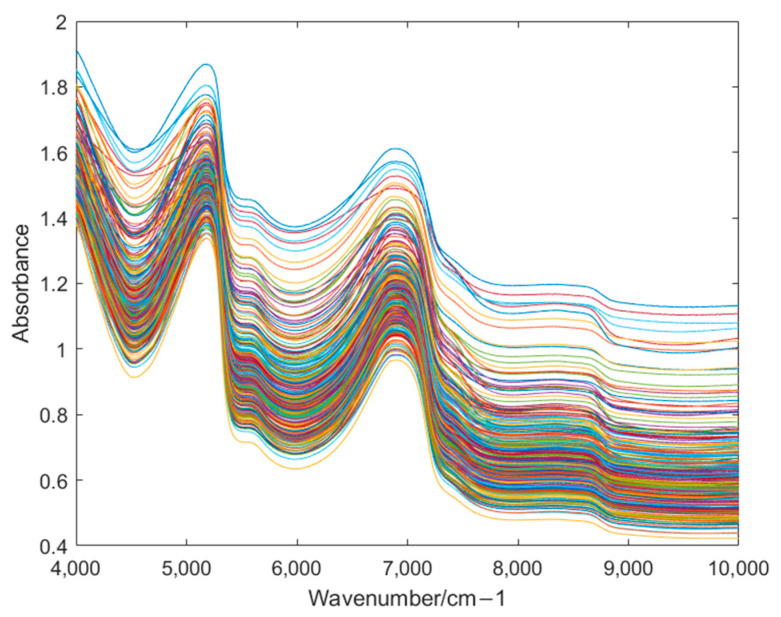
The original NIR spectra of lettuce samples.

In this study, NIR spectra of lettuce samples with five different storage times were obtained. As shown in Figure 2, the spectral wavenumber range was 4000–10,000 cm^−1^. NIR spectra contained a large number of molecular chemical bonds and functional group vibration characteristics, namely the absorption bands in the spectra. These vibration characteristics could be used to identify the chemical bonds and functional groups in the samples and further analyze the molecular structure and chemical composition of the samples [51,52]. However, not all wavenumber points in the spectral data contain effective information. Therefore, it is very important to select the appropriate wavenumber in the NIR spectra. This not only avoids invalid redundant information but also quickly locates the effective absorption bands to accurately extract the characteristic information of the samples. As shown in Figure 2, there are two absorption bands in the spectra collected in the range of 4500–5200 cm^−1^ and 6000–7000 cm^−1^. The absorption band near 5100 cm^−1^ is related to the second harmonic and combination vibration of the O-H bond in water in lettuce [53]. During storage, the water content in lettuce decreases with the extension of storage time, which leads to the weakening of the absorption band intensity near 5100 cm^−1^ [54]. The absorption band in the range of 6000–7000 cm^−1^ is related to the first overtone of the N-H stretching vibration of CONH [55], and its intensity may decrease with the degradation of protein during the storage of lettuce.

### 3.2. Preprocessing of NIR Spectra

**Figure 3:**
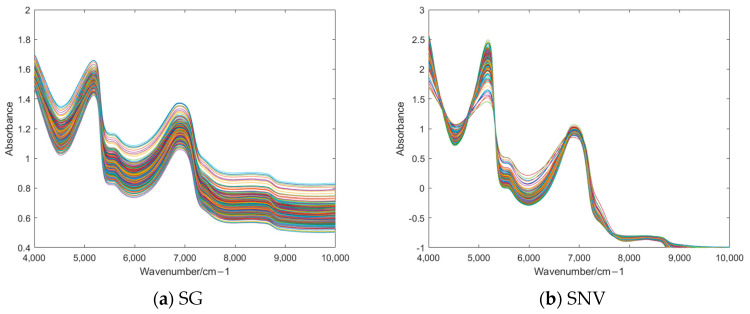

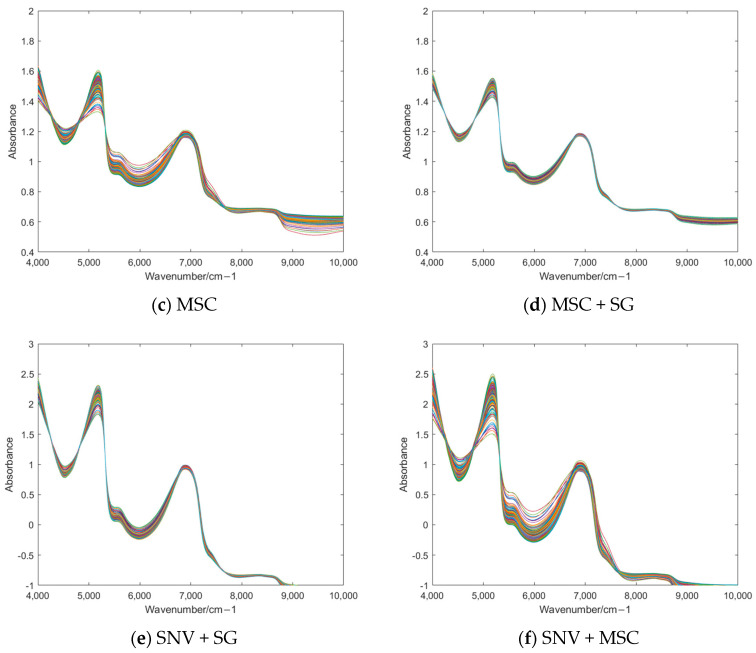
NIR spectra after different preprocessing methods. SG, Savitzky–Golay; SNV, standard normal variate; MSC, multiplicative scatter correction; MSC + SG, multiplicative scatter correction and Savitzky–Golay; SNV + SG, standard normal variate and Savitzky–Golay; SNV + MSC, standard normal variate and multiplicative scatter correction.

Figure 3 illustrates the NIR spectra of lettuce samples processed by different preprocessing methods. Six preprocessing methods, i.e., SG, SNV, MSC, MSC + SG, SNV + SG, and SNV + MSC, were adopted to process the NIR spectra. As shown in Figure 3, after the NIR spectra were preprocessed using SNV, MSC, SNV + SG, and SNV + MSC, the spectral absorption band features became more obvious, reducing the differences between samples and enhancing the comparability. These four preprocessing methods were the most effective. However, it cannot be determined solely from the spectra. It is necessary to combine feature extraction and compare the classification accuracy to confirm the best preprocessing method.

After preprocessing, feature extraction was made using ALDA, CLDA, MLDA, and NLDA, as shown in Table 1 and Table 2. Although the average classification accuracy of SNV-CatBoost was the highest, the MSC-NLDA-CatBoost model had the highest classification accuracy, reaching 97.67%. Therefore, by comparing the preprocessed NIR spectra and the classification accuracy after feature extraction, MSC is the best preprocessing method for the NIR spectra of lettuce.

### 3.3. Feature Extraction

After the NIR spectra of lettuce were preprocessed using MSC, four algorithms, namely ALDA, CLDA, MLDA, and NLDA, were, respectively, adopted to perform feature extraction from the preprocessed spectra. Applying the above four methods to the training set, respectively, the feature vectors were calculated, and the total samples were projected onto the four eigenvectors corresponding to the largest eigenvalues. This process simplifies the data into four dimensions, thereby achieving the effect that within the same category sample set, samples of different categories are dispersed.

#### 3.3.1. Feature Extraction Using ALDA

**Figure 4:**
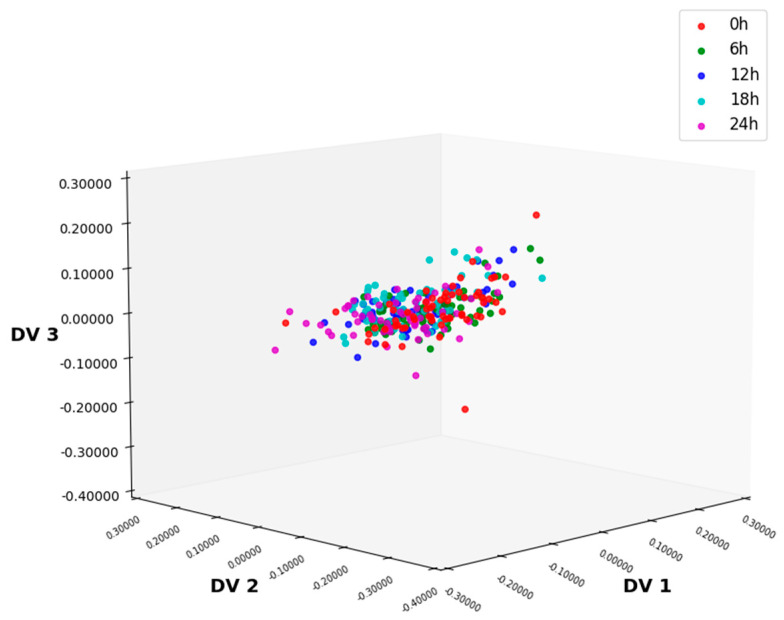
Distribution of 3D data after ALDA processing under MSC preprocessing methods. ALDA, approximate linear discriminant analysis; MSC, multiplicative scatter correction.

ALDA achieves the extraction of feature information from the training set samples by approximating the intra-class scatter matrix of the sample data. The three-dimensional data distribution after feature extraction via ALDA is shown in Figure 4. The distribution of sample points is chaotic and completely without regularity. Additionally, there is overlap between categories, and the sample points of each category are not concentrated, making it impossible to accurately classify the samples of each category. The classification accuracy of MSC-ALDA is 38.67%. Thus, the combination of MSC preprocessing and ALDA cannot accurately classify lettuce samples with different storage times.

#### 3.3.2. Feature Extraction Using CLDA

**Figure 5:**
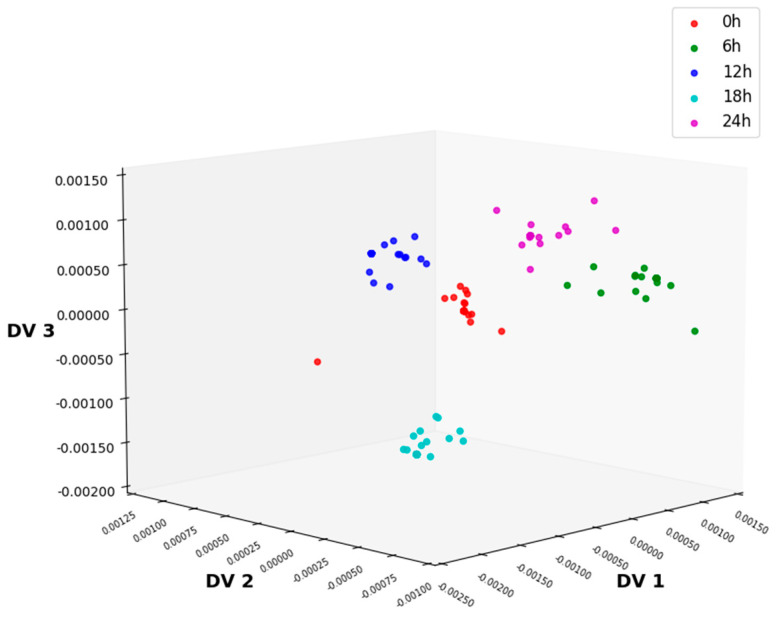
Distribution of 3D data after CLDA processing under MSC preprocessing methods. CLDA, common-vector linear discriminant analysis; MSC, multiplicative scatter correction.

Figure 5 shows the three-dimensional data processed via MSC-CLDA. CLDA extracts feature information from the training samples and obtains the common vectors. As shown in Figure 5, after the feature extraction using CLDA, the boundaries between different categories of sample data are obvious, while the samples of the same category are relatively concentrated. Compared with ALDA, the CLDA algorithm achieves a higher classification accuracy rate of 96.67%, effectively and accurately distinguishing lettuce samples with different storage times.

#### 3.3.3. Feature Extraction Using MLDA

**Figure 6:**
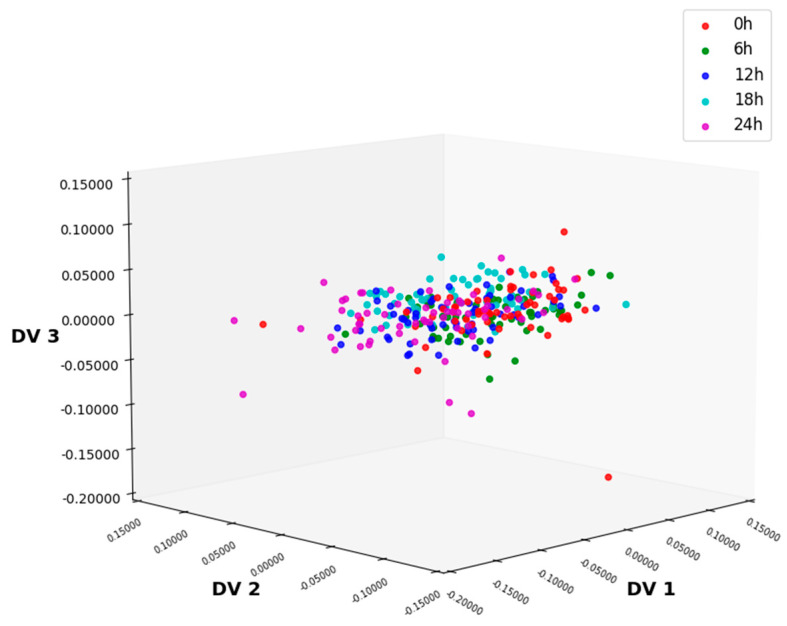
Distribution of 3D data after MLDA processing under MSC preprocessing methods. MLDA, maximum-uncertainty linear discriminant analysis; MSC, multiplicative scatter correction.

The distribution of the NIR spectra after feature extraction using the MLDA algorithm is shown in Figure 6. MLDA solves the SSS problem by increasing the eigenvalues of the within-class scatter matrix and extracting the feature information of the lettuce samples. Similar to ALDA, the data distribution of the MSC-MLDA model is chaotic and disorderly, and there is overlap among different categories of samples. From the data distribution graph, it can be seen that the feature extraction effect obtained using MSC combined with MLDA is poor, and the distribution of sample points of each category is too scattered, which is not suitable for accurate classification between different categories of samples. Its classification accuracy is only 46.67%.

#### 3.3.4. Feature Extraction Using NLDA

**Figure 7:**
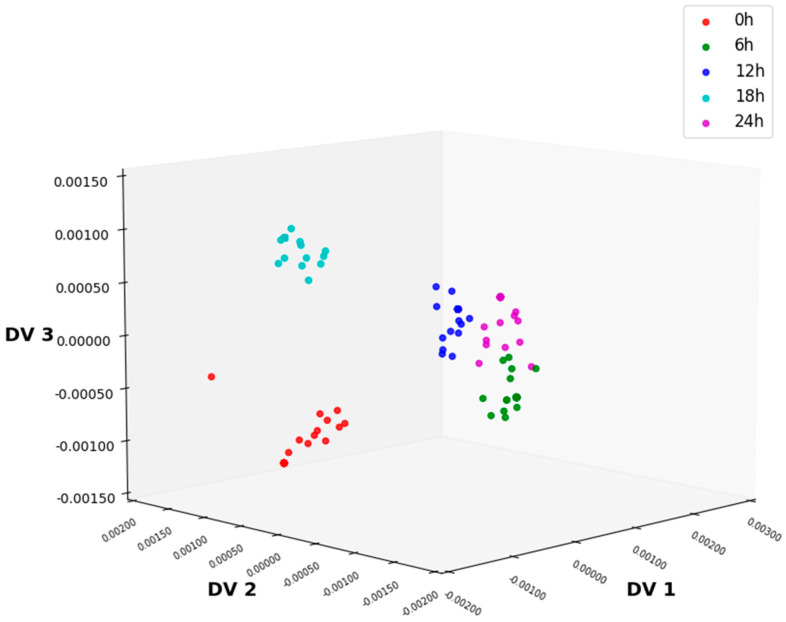
Distribution of 3D data after NLDA processing under MSC preprocessing conditions. NLDA, null-space linear discriminant analysis; MSC, multiplicative scatter correction.

NLDA eliminates intra-class differences by projecting the training data onto the null space of the intra-class scatter matrix and extracts the feature information of the sample data by solving the eigenvectors of the inter-class scatter matrix. Projecting the data onto the eigenvectors yields the reduced-dimensional data distribution, as shown in Figure 7. Compared with data distributions processed via ALDA and MLDA, NLDA yields significantly clearer boundaries between different classes, facilitating more accurate classification. Compared with CLDA, although the 6 h and 24 h data have a slight overlap, the distribution of samples in different classes is more concentrated. Its classification accuracy is the highest, reaching 97.67%, indicating that MSC-NLDA can achieve accurate classification of lettuce samples with different storage times.

### 3.4. Classification Results

After preprocessing and feature extraction operations on the NIR spectra of lettuce samples, a classifier is required to conduct discriminant analysis for different storage times of lettuce samples. In this experiment, two classifier algorithms, namely CART and CatBoost, were adopted to realize the classification task. After preprocessing and feature extraction, the above two classification models were trained using the training samples and sample categories. Then, the classification models were performed to predict the categories of the test samples. The predicted results were compared with the original test sample categories to determine the classification accuracy of the model. At last, the best classification model was determined based on the final classification accuracy.

#### 3.4.1. Classification Using CART

In this experiment, we first applied the CART classifier to discriminate the data after feature extraction. The classification accuracies are shown in Table 1. When NLDA was combined with the CART classifier, the classification accuracy exceeded 74% in both cases. This indicated that NLDA was significantly superior to other feature extraction algorithms. Especially for the SNV-NLDA combined with the CART, the classification accuracy reached 91%. The CART model can achieve an accurate classification of the lettuce storage time, but there is still further improvement in the model’s performance.

#### 3.4.2. Classification Using CatBoost

Considering that CART is a single decision tree algorithm, its generalization ability is relatively weak, the model complexity is limited, and it is susceptible to noise. In this experiment, we attempted to use the CatBoost algorithm to establish a classification model and compared the classification results with CART to confirm the final classification model. The classification results of the CatBoost model are shown in Table 2. The overall average classification accuracy of CatBoost was significantly higher than that of CART. When NLDA was combined with CatBoost, the classification accuracy was above 86%, and the highest classification accuracy reached 97.67%, which was significantly better than CART in classification. Therefore, NLDA was superior to other feature extraction algorithms. Moreover, compared with CART, the classification performance of the CatBoost model was better and the classification accuracy was higher. Therefore, NLDA combined with CatBoost is the optimal classification model for lettuce with different storage times.

## 4. Discussion

In this study, firstly, the NIR spectrum data of lettuce samples were collected using an NIR spectrometer, and six kinds of preprocessing methods were, respectively, performed on the spectral data. As shown in Figure 2 and Figure 3, MSC could significantly improve the degree of absorption bands in the spectral data and enhance the comparability among the spectra of different types of samples. After preprocessing, feature extraction was carried out on the sample data. In this experiment, four feature extraction algorithms, namely ALDA, CLDA, MLDA, and NLDA, were adopted. As shown in Figure 4, Figure 5, Figure 6 and Figure 7, NLDA effectively distinguishes different types of samples, yielding clear classification boundaries and concentrating same-category sample data in a single location. After feature extraction processing, classification was carried out on the test data using two classification algorithms, namely CART and CatBoost. The classification results are shown in Table 1 and Table 2. After comparison, the classification accuracy of CatBoost was higher than that of CART. Thus, CatBoost has the best classification performance, especially the MSC-NLDA-CatBoost classification model, whose classification accuracy could reach 97.67%. Therefore, the classification model constructed by feature extraction combined with CatBoost can achieve an accurate classification of lettuce storage times.

**Figure 8:**
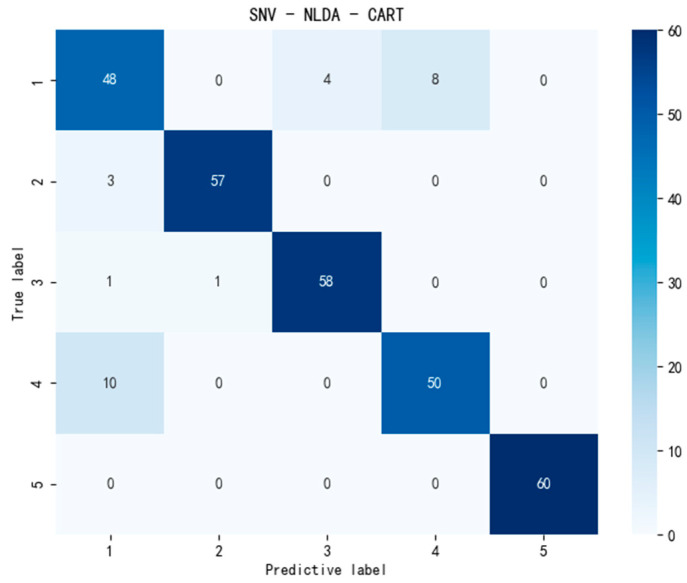
Confusion matrix of the SNV-NLDA-CART model. SNV-NLDA-CART, standard normal variate, and null-space linear discriminant analysis and classification and regression trees.

**Figure 9:**
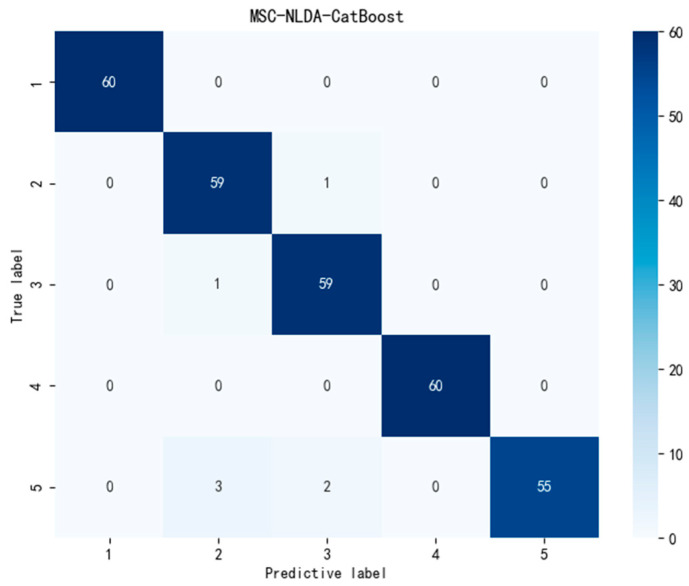
Confusion matrix of the MSC-NLDA-CatBoost model. MSC-NLDA-CatBoost, multiplicative scatter correction, and null-space linear discriminant analysis and categorical boosting.

However, evaluating model performance solely based on classification accuracy lacks reliability. It is necessary to combine multiple indicators to comprehensively verify the generalization ability and stability of the model. Therefore, in addition to comparing classification accuracy, this experiment introduced confusion matrix diagrams to more intuitively compare the performances of the CART and CatBoost models. The highest classification accuracy of the CART model could reach 91%, and that of the CatBoost model could achieve 97.67%. Therefore, we drew the confusion matrix diagrams of the two optimal models, SNV-NLDA-CART and MSC-NLDA-CatBoost, for comparative analysis, as shown in Figure 8 and Figure 9. Based on the confusion matrixes, this study further calculated the precision, recall, and F1 score of each model. The matrixes were used to more comprehensively evaluate the category prediction ability and overall balance ability of the models. In addition, this study also calculated the standard deviation of the classification accuracy of each model to assess the stability of the classification model. The evaluation indicators of each model are shown in Table 3.

As shown in Figure 8 and Figure 9 and Table 3, the CatBoost classification model can accurately predict the category of samples and outperforms CART in various model evaluation metrics. This is because the CART model consists of only a single decision tree, which has relatively poor generalization ability, resulting in its model performance being inferior to that of CatBoost. Since CatBoost forms a strong classifier by training multiple weak classifiers, the generalization ability of the CatBoost model is stronger. Additionally, due to its unique feature processing method and ordered boosting process, the category prediction ability, overall balance ability, and stability of the CatBoost model are all superior to those of CART. Therefore, the combination of feature extraction and CatBoost can achieve rapid and accurate classification of the lettuce storage time.

As shown in Table 3, the classification models constructed by combining MSC-NLDA with CatBoost achieved the best performance in all evaluation metrics. The classification accuracy, precision, and recall all reached approximately 97.7%, and the F1 score reached 0.98. Moreover, the standard deviation of the accuracy was 0.017. These results prove that the MSC-NLDA-CatBoost models are the best classification models and can accurately classify lettuce samples with different storage times. They also demonstrate high reliability and stability in practical applications, enabling rapid and precise prediction of the storage status of lettuce samples and their classification. The summary of the best models is shown in Table 4, and the best comprehensive indicators are presented in the second column of Table 3.

In the current classification models based on NIR spectroscopy for lettuce storage time, in terms of feature extraction, the NLDA applied in this experiment can effectively solve the SSS problem in practical applications and avoid the loss of discriminative information, compared with the traditional combination of PCA and LDA. In terms of classification algorithms, CatBoost has a stronger discriminative ability and generalization ability than the traditional KNN. Therefore, compared with other current classification models, the combination of feature extraction and CatBoost has significant advantages in multiple key performance indicators, with higher classification accuracy, reliability, and stability.

However, due to the single category of lettuce samples and the limited sample size in this experiment, the application scope of the model in practical applications may be limited. Moreover, when dealing with large-scale datasets, since there is a lack of spectral wavenumber selection processing, the computational efficiency and cost consumption still need to be further evaluated. Initially, in this experiment, a successive projection algorithm (SPA) wavenumber selection operation was added after the preprocessing operation. However, since the feature extraction methods in this experiment are more suitable for dealing with the SSS problem, while the data sample dimensionality decreases after SPA processing, the SSS problem does not exist and the classification accuracy after the feature extraction processing decreases instead. Especially for NLDA, it is impossible to extract effective features after SPA, resulting in a classification accuracy rate of 0%. The specific results are shown in Table 5. Therefore, in subsequent research, the universality and reliability of the model will be further verified. Other wavenumber selection algorithms will be attempted to improve the computational efficiency of the model. The model will be improved and optimized according to the actual application situation.

## 5. Conclusions

This experiment constructed a classification model based on feature extraction algorithms and the CatBoost algorithm to identify the lettuce storage time. At first, the NIR spectra of lettuce samples were obtained with an NIR spectrometer. Next, the spectral data were processed using preprocessing algorithms (SG, SNV, MSC, MSC + SG, SNV + SG, and SNV + MSC). Then, the ALDA, CLDA, MLDA, and NLDA algorithms were used to extract features and reduce the dimensionality of the sample data. Lastly, the CART and CatBoost algorithms were employed to classify the data. Furthermore, by comparing the classification accuracy of different models, the best classification model was determined.

The experimental results indicate that combining CatBoost with feature extraction yields superior classification performance compared to CART. The combination of NLDA and CatBoost to build a classification model, along with NIR spectroscopy, can achieve a non-destructive, rapid, and accurate prediction of lettuce storage time.

However, in this study, the proposed classification model has only been applied and verified for Italian all-year-round bolt-resistant lettuces. The classification prediction effect for other lettuce varieties still needs further verification. Additionally, the sample size in this experiment is limited, which to some extent restricts the universality and reliability of the model. Therefore, the classification model proposed in this study has certain limitations in wide application.

In subsequent research, we will expand the variety range of lettuce samples and increase the sample size to enhance the representativeness and generalization ability of the classification model. We will further improve and optimize the model performance based on its effect in practical application to ensure it can accurately and non-destructively analyze the storage status of different types of lettuce. Moreover, we will introduce an external validation mechanism to comprehensively evaluate the stability and universality of the model, thereby further improving it.

## Figures and Tables

**Table 1:** Classification accuracy rates of preprocessing methods combined with feature extraction methods under the CART algorithm (%).

Feature Processing	SG	SNV	MSC	MSC + SG	SNV + SG	SNV + MSC
ALDA	54	33.67	35.67	54	56.33	35.67
CLDA	47.67	83	82.33	47.67	48.67	82.33
MLDA	46	36	37.33	60.33	60	37.33
NLDA	89.67	91	88	74.67	75.33	89.67
Average accuracy rate	59.34	60.92	60.83	59.17	60.08	61.25

Abbreviations: ALDA, approximate linear discriminant analysis; CLDA, common-vector linear discriminant analysis; MLDA, maximum-uncertainty linear discriminant analysis; NLDA, null-space linear discriminant analysis; SG, Savitzky–Golay; SNV, standard normal variate; MSC, multiplicative scatter correction.

**Table 2:** Classification accuracy rates of preprocessing methods combined with feature extraction methods under the CatBoost model (%).

Feature Processing	SG	SNV	MSC	MSC + SG	SNV + SG	SNV + MSC
ALDA	56.33	37.33	38.67	58.33	55.67	38.67
CLDA	59	95.67	96.67	62.33	60.33	95.33
MLDA	53	51.33	46.67	64.67	63	46.33
NLDA	95.33	96	97.67	86.67	86	95.33
Average accuracy rate	65.92	70.08	69.92	68	66.25	68.92

Abbreviations: ALDA, approximate linear discriminant analysis; CLDA, common-vector linear discriminant analysis; MLDA, maximum-uncertainty linear discriminant analysis; NLDA, null-space linear discriminant analysis; SG, Savitzky–Golay; SNV, standard normal variate; MSC, multiplicative scatter correction.

**Table 3:** The evaluation indicators of the CART and CatBoost models.

Evaluation Indicators	Accuracy Rate (%)	Precision (%)	Recall (%)	F1 Score	Standard Deviation
SNV-NLDA-CART	91	91.74	91	0.9088	0.0389
MSC-NLDA-CatBoost	97.67	97.84	97.67	0.9766	0.017

Abbreviations: SNV-NLDA-CART, standard normal variate and null-space linear discriminant analysis and classification and regression trees; MSC-NLDA-CatBoost, multiplicative scatter correction and null-space linear discriminant analysis and categorical boosting.

**Table 4:** The best models at each stage.

Stage	Method
Preprocessing	multiplicative scatter correction
Feature extraction	null-space linear discriminant analysis
Classification	categorical boosting

**Table 5:** Classification accuracy rates of preprocessing methods combined with feature extraction methods and the CatBoost model using SPA (%).

Feature Processing	SG	SNV	MSC	MSC + SG	SNV + SG	SNV + MSC
ALDA	53.67	45	45	71	64.67	39.33
CLDA	41.67	27	26.33	48.33	43.67	31.67
MLDA	50.67	39.33	38.33	72	65.33	36.67
NLDA	0	0	0	0	0	0
Average accuracy rate	36.5	27.83	27.42	47.83	43.42	26.92

Abbreviations: ALDA, approximate linear discriminant analysis; CLDA, common-vector linear discriminant analysis; MLDA, maximum-uncertainty linear discriminant analysis; NLDA, null-space linear discriminant analysis; SG, Savitzky–Golay; SNV, standard normal variate; MSC, multiplicative scatter correction; SPA, successive projections algorithm.

## Data Availability

The original contributions presented in the study are included in the article, further inquiries can be directed to the corresponding authors.
